# Organosilica-Modified Multiblock Copolymers for Membrane Gas Separation

**DOI:** 10.3390/polym13203579

**Published:** 2021-10-17

**Authors:** Ilsiya M. Davletbaeva, Alexander Yu. Alentiev, Zulfiya Z. Faizulina, Ilnaz I. Zaripov, Roman Yu. Nikiforov, Victor V. Parfenov, Alexander V. Arkhipov

**Affiliations:** 1 Department of Technology of Synthetic Rubber, Kazan National Research Technological University, 68 Karl Marx str, 420015 Kazan, Russia; faizulina.alin@yandex.ru (Z.Z.F.); zaripovilnaz@gmail.com (I.I.Z.); 2 A.V. Topchiev Institute of Petrochemical Synthesis of Russian Academy of Sciences, 29 Leninsky Prospect, 119991 Moscow, Russia; alentiev1963@mail.ru (A.Y.A.); nru@ips.ac.ru (R.Y.N.); 3 SIBUR LLC, 16, bld.3, Krzhizhanovskogo Str., GSP-7, 117997 Moscow, Russia; 4 Department of Solid State Physics, Kazan Federal University, 18 Kremlyovskaya Str, 420008 Kazan, Russia; victor.parfenov@kpfu.ru; 5 Institute of Electronics and Telecommunications, Peter the Great St.Petersburg Polytechnic University, 29 Polytechnicheskaya st., 195251 St. Petersburg, Russia; Arkhipov@rphf.spbstu.ru

**Keywords:** macroinitiator, 2,4-toluene diisocyanate, organosubstituted silica derivatives, modification, multiblock copolymers, polymer membranes, gas permeability

## Abstract

Organosubstituted silica derivatives were synthesized and investigated as modifiers of block copolymers based on macroinitiator and 2,4-toluene diisocyanate. A peculiarity of the modified block copolymers is the existence in their structure of coplanar rigid polyisocyanate blocks of acetal nature (O-polyisocyanates). Organosubstituted silica derivatives have a non-additive effect on high-temperature relaxation and α-transitions of modified polymers and exhibit the ability to influence the supramolecular structure of block copolymers. The use of the developed modifiers leads to a change in the gas transport properties of block copolymers. The increase of the permeability coefficients is due to the increase of the diffusion coefficients. At the same time, the gas solubility coefficients do not change. An increase in the ideal selectivity for a number of gas pairs is observed. An increase in the selectivity for the CO_2_/N_2_ gas pair (from 25 to 39) by 1.5 times demonstrates the promising use of this material for flue gases separation.

## 1. Introduction

Nowadays, multiblock copolymers (MBCs) capable of forming a wide variety of supramolecular structures are of great interest to researchers [1,2,3,4]. The chemical structure of MBC can be designed a way that interactions between materials of different chemical nature, polarity and binding energy can be largely controlled by their modification. Segments combination of block copolymers differing in chemical structure has great potential for creating new supramolecular structures and controlling their morphology [5]. Chain growth mechanisms are under special attention by developing methods for the production of new multiblock copolymers [6,7]. The simplest method of multiblock copolymers preparation is in the usage of macromonomers or macroinitiators in conventional stepwise growth polymerization [8,9]. Multiblock copolymers can be used as structural materials, adhesives, emulsifiers, modifiers, drug-delivery materials and gas-transport membranes [10,11,12,13,14]. 

MBCs containing polyester or polyethylene oxide (PEO) blocks are interesting in the production of gas transport membranes. In Reference [15], the properties of a poly(styrene–ethylene oxide–styrene) triblock copolymer and its mixture with polyethylene glycol as a membrane for CO_2_ evolution were investigated. The design of the CO_2_ selective membranes was based on the unusually high affinity of CO_2_ to polyethylene oxide blocks. As a result, CO_2_ permeability was increased by adding PEO to the membrane composition. A similar poly(styrene–ethylene oxide–styrene) was synthesized, but differing in the content of the crystalline segment of PEO [16]. It was shown that the ratio of the crystalline and amorphous phases in the polymer can be optimized by changing the PEO content and the selectivity to pair CO_2_/gas could be significantly improved by changing the morphology of triblock copolymers.

Comparison of existing membranes based on block and random copolymers obtained by use PEG was carried out [17]. An increase of the PEG content in membranes decreases the gas permeability coefficient. The selectivity in this case increases, reaching the values α = 37.31 for the CO_2_/CH_4_ pair and α = 33.44 for H_2_/CH_4_ pair at 20% PEG content.

The study’s results of the dependence between the structure and gas transport characteristics of polyurethane films obtained from simple and complex polyesters are presented [18,19]. The permeability of gases through polyurethane-based membranes can also be controlled by changing the ratio of rigid and flexible segments [20,21]. Usually, the mechanical properties of polyurethanes improve with an increase in the content of rigid segments, but their gas permeability decreases [22,23]. This is due to a decrease in both the free volume and an increase in the content of rigid blocks having a higher glass transition temperature than flexible segments [24,25,26]. Polyurethanes with a higher glass transition temperature have less segmental mobility and therefore have a lower diffusion. The rigid segments act as particles filler and crosslinking agent that restrain the movement of the elastic segments and lead to decrease of gas permeability. On the contrary, an increase of flexible segments content in polyurethanes leads to the perfection of microphase separation and an increase their gas permeability [27,28]. A series of polyurethane-urea membranes containing polyester fragments [26] were synthesized, and polyurethane membranes containing ether–siloxane segments, promising for the separation of n-C_4_H_10_/CH_4_ mixtures, were investigated [29]. The nature of the flexible segments of polyurethanes affects the efficiency of transporting gas across membranes [30,31]. For polyurethane membranes based on polyoxyethylene glycol, high selectivity, but low gas permeability to CO_2_ was observed [32]. However, the practical application of membranes based on polyurethane is limited by their poor thermomechanical stability and their low resistance to plasticization during gas separation.

The basis for the construction of thermodynamically stable hybrid polymer materials with a nanoscale structure can be their modification [33,34,35]. The addition of nanoparticles affects permeability, selectivity, hydrophilicity, conductivity, mechanical strength and thermal stability [36]. Hybrid membranes were obtained by introducing tetramethylorthosilicate into polyamic acid in the presence of dimethylacetamide [37,38]. The synthesized hybrid membrane has a higher permeability, selectivity and solubility for CO_2_. Polyimide-siloxane hybrid membranes were obtained by polycondensation, imidization and a sol–gel process [39]. Aminopropyltrimethoxysilane and aminopropylmethyldiethoxysilane were used as binding agents. Gas permeability increased with the introduction of siloxane and higher rates were observed when using aminopropyltrimethoxysilane as a binder. SiO_2_ was used as a filler in polyimide hybrid membranes for CO_2_/CH_4_ separation [40]. Silica in various volume fractions was also used as a filler for polysulfone membranes [41,42]. Membranes filled with nanoparticles exhibit increased thermal stability and increased CO_2_ permeability with increasing SiO_2_ content. SiO_2_ particles lead to the destruction of polymer chains and a subsequent increase in free volume. 

**Scheme 1:**
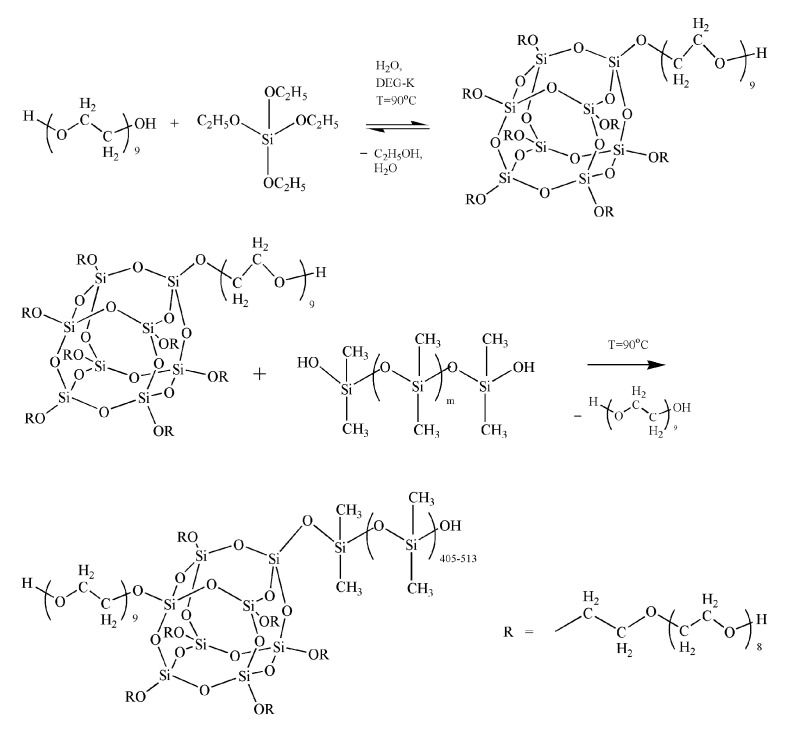
ASiP synthesis.

Earlier, silica branched by polyoxyethylene oxide and low-molecular-weight polydimethylsiloxane (Scheme 1) were obtained [43].

Silica branched by polyoxyethylene oxide (PEOASiP) was obtained at the first stage. In the second stage, the ASiP was synthesized as a result of the transetherification reaction based on the PEOASiP and low-molecular-weight polydimethylsiloxane.

**Scheme 2:**
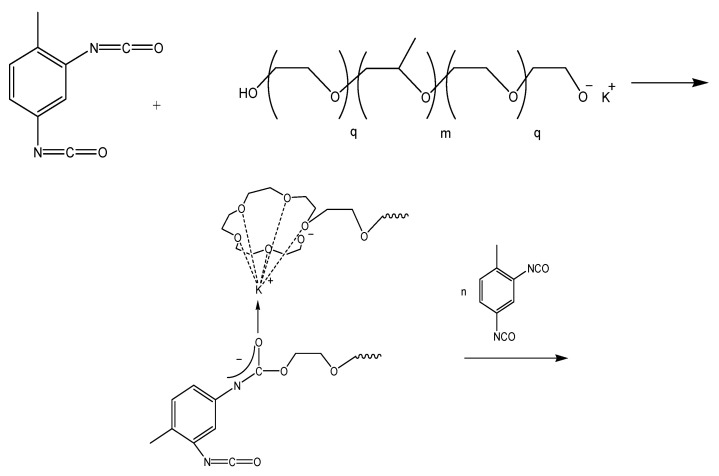

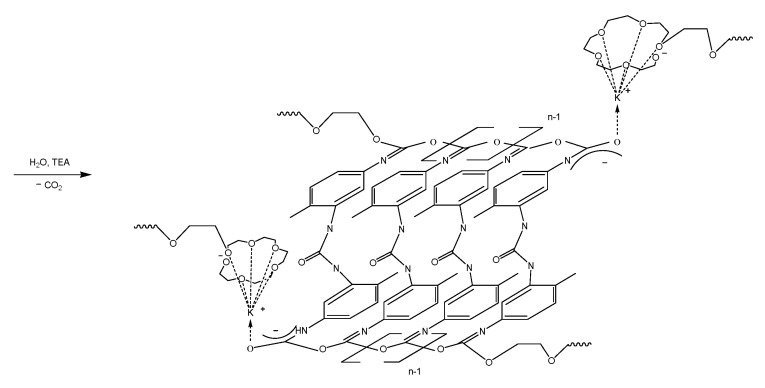
O-polyisocyanate blocks formation.

It was found that the ASiP has a significant effect on the processes of block copolymers microphase separation. Block copolymers were prepared by using 2,4-toluene diisocyanate (TDI) and copolymers of ethylene and propylene oxides terminated with potassium alcoholate groups (PPEG, MW = 4200). These polymers are of interest due to their high gas transmission characteristics [11,12]. The peculiarity of their supramolecular structure is due to the formation of coplanar rigid polyisocyanate blocks of acetal nature (O-polyisocyanates) (Scheme 2). O-polyisocyanate blocks, polyoxyethylene and polyoxypropylene blocks of PPEG participate in the formation of the supramolecular structure [44].

In this work, changes were made to the structure of the PEOASiP by replacing a part of tetraethoxysilane (TEOS) with 3-aminopropyltriethoxysilane (AGM) in order to incorporate functionalized group to the polymer. Partial replacement of TEOS with AGM-9 in the synthesis of silica branched by polyoxyethylene oxide is associated with the need to enhance their amphiphilicity. The structure and content effect of the developed modifiers on the supramolecular organization and gas transport characteristics of block copolymers containing coplanar O-polyisocyanate blocks were studied.

## 2. Materials and Methods

### 2.1. Materials 

Polyoxyethylene oxide H(O–CH_2_–CH_2_)_9_–OH (PEO, molecular weight is 400 g/mol) was purchased from PJSC Nizhnekamskneftekhim (Nizhnekamsk, Russia), tetraethoxysilane (TEOS) was purchased from CJSC Vekton (St. Petersburg, Russia). Potassium diethylene glycolate (DEG-K) with formula H(O–CH_2_–CH_2_)_2_–OK was obtained in laboratory conditions. The block copolymer of propylene and ethylene oxide (PPEG) with formula HO[CH_2_CH_2_O]_n_[CH_2_(CH_3_)CH_2_O]_m_[CH_2_CH_2_O]_n_K, where n ≈ 14 and m ≈ 48, molecular weight 4200 g/mol, containing 30 wt.% of peripheral polyoxyethylene blocks, where the content of potassium alcoholate groups is 10.9% from the total number of functional groups, was purchased from PJSC Nizhnekamskneftekhim (Nizhnekamsk, Russia). We purchased 2,4-toluene diisocyanate 98% (TDI) and 3-aminopropyltriethoxysilane (AGM) from Sigma-Aldrich (St. Louis, MO, USA). Polydimethylsiloxane (PDMS, average molecular weight is 30,000 g/mol) was purchased from JSC Kazan factory of Synthetic Rubber (Kazan, Russia).

PEO and PPEG were additionally dried at reduced pressure (approximately 0.1 kPa) and at elevated temperature of 95 °C down to 0.01 wt.% moisture concentration. 

### 2.2. Synthesis of the ASiP–AGM/the ASiP

The synthesis of the ASiP and the ASiP–AGM was multistage. At the first stage, PEO (9.64 mL), TEOS (20 wt.% of the total amount of oligomers and TEOS, 4.76 mL), water (0.04 wt.% of the total weight of components PEG–TEOS) and DEG-K catalyst (0.10 wt.% relative to the mass of the total reaction system, 20 µL) were added to the flask. The mixtures were stirred at temperature 90 °C for at least 6 h. Transetherification of ethoxysilane. 

TEOS’s groups, sequential reactions of their hydrolysis with condensation of the resulting Si–OH groups simultaneously proceeded in the DEG-K presence and water. The second part of TEOS (10 wt.% by weight of the total amount of TEOS, 2.38 mL) was introduced into the reaction system. The synthesis was carried out for 6 h, after the final part of TEOS (5 wt.% by weight of the total number of TEOS, 1.4 mL) was introduced in the mixture with stirring for 4 h. After each stage, mixtures were put in vacuum for 10 min to remove alcohol and water and shift the balance towards the product. As a result of the described reaction processes, including the interaction of PEO, TEOS with a catalyst.

DEG-K and water, was obtained silica branched by polyoxyethylene oxide (PEOASiP). Further, PDMS (5 mL) was added in the PEOASiP with stirring at temperature 90 °C, the viscosity increased in reaction system and the ASiP was obtained. 

The synthesis of the ASiP–AGM is similar to the PEOASiP synthesis. The difference is that AGM (10 ÷ 100 wt.% of the total number of TEOS) was added in the PEOASiP with stirring for 4 h, at a temperature 90 °C, after the viscosity increased in reaction system and the ASiP–AGM-(10 ÷ 100) was obtained.

### 2.3. Synthesis of Modified by the ASiP–AGM/ASiP Multiblock Copolymers Based on PPEG and TDI 

The reaction was carried out in toluene at 20 °C in a flask equipped with a reflux condenser. The polymerization proceeded with the constant stirring. PPEG (1 g), toluene (7.9 g), bisphenol A (0.004 g), acetic acid (0.007 g) and calculated amount of the ASiP–AGM/the ASiP were added to the flask. The reaction mass was mixed at a given temperature until the complete dissolution of PPEG took place, then TDI (0.62 g.) at a molar ratio [PPEG]: [TDI] = 1:15 was introduced. Five minutes after mixing with TDI, triethylamine (0.0072 g) and water (0.001 g) were loaded into the reaction system. After ten minutes, the reaction mass was dispensed to Petri dish, and it was cured at room temperature during 72 h. The final polymer films swell in acetone and water.

### 2.4. Methods

#### 2.4.1. Fourier Transform Infrared Spectroscopy Analysis (FTIR)

The FTIR spectra were recorded on an InfraLUM FT 08 Fourier transform spectrometer (Lumex, St. Petersburg, Russia), using the attenuated total reflection technique in the spectral range of 3800–600 cm^−1^. The spectral resolution was 2 cm^−1^ and the number of scans was 16.

#### 2.4.2. Thermal Gravimetric Analysis (TGA)

TGA was performed using STA-600 TGA–DTA combined thermal analyzer (PerkinElmer, Waltham, MA, USA). The samples (0.1 g) were loaded in alumina pans and heated from 30 to 430 °C, at a rate of 5 K/min, in a nitrogen atmosphere.

#### 2.4.3. Light-Scattering (DLS)

Dynamic light scattering experiments were carried out on Zetasizer Nano ZS (Malvern, UK). This instrument has 4 mV He–Ne laser working on 632.8 nm wavelength. Measurements were carried out at 173° detection angle. The experiments were carried out at 25 °C in the disposable plastic cuvettes of 1 cm path length.

#### 2.4.4. Tensile Stress–Strain Measurements

Tensile stress–strain measurements were obtained from the film samples of size 40 mm × 15 mm with Universal Testing Machine Inspekt mini (Hegewald&PeschkeMeß-und Prüftechnik GmbH, Nossen, Germany) at 293 ± 2 K, 1 kN. The crosshead speed was set at 50 mm/min and the test continued until sample failure. A minimum of five tests were analyzed for each sample and the average values were reported. 

#### 2.4.5. Electron Spectroscopy

Sorption of Rhodamine 6G on polymer films was carried out by keeping the polymers in alcohol solutions of Rhodamine 6G (0.005 mol/L) for 10 s at room temperature. The film was washed in distilled water and dried after removing Rhodamine 6G. Dry films were secured in a holder of the device.

Film samples were studied by using a UV 1800 double-beam scanning spectrophotometer (Zhimadzu, Kyoto, Japan) on a silicon photodiode in the UV range. The spectral range of the apparatus is 190–1100 nm, and the wavelength reproducibility is ±0.1 nm.

#### 2.4.6. Measurements of the Surface Tension

The droplet counting method was used to determine the surface tension (σ). The basis of the calculations is the law, where the weight of the drop that comes off the pipette is proportional to the surface tension of the fluid and the radius of the pipette (R): *m* = *2π∙R∙σ/g*, where *g* is acceleration of gravity, and *m* is the dropmass of the test liquid. Following this equation is *σ* = *m∙g/2π∙R*. Further, according to the obtained results, a characteristic curve of the surface tension (*σ*) from concentration (*C*) was constructed.

#### 2.4.7. Measurement of Temperature Dependence of Dielectric Loss Tangent (DLT)

The temperature dependence of the DLT of polymer samples (thickness of 0.5–0.7 mm) was registered in the temperature range from −120 to 130 °C at a frequency of 1 kHz. A facility consisted of the measuring cell that was placed in a Dewar vessel filled with nitrogen, and to which an E7–20 RLC-meter and a B7–78 universal voltmeter functioning as a precision thermometer were connected.

#### 2.4.8. Permeability Measurements

The permeability coefficients of H_2_, He, N_2_, O_2_, CO_2_ and CH_4_ and diffusion coefficients of N_2_, O_2_, CO_2_ and CH_4_ gases for block-copolymer film samples (120–200 mkm thickness) were obtained by the integral barometric method on the MKS Barotron installation at room temperature (23 ± 1 °C); pressure above the membrane was 1–5 bar, pressure below the membrane was ~0.16 mbar. The permeability coefficients were determined from the steady-state gas permeation experiment. The permeability coefficient is represented in extra-system Barrer unit (1 Barrer = 3.348 10^−16^ mol m m^−2^ s^−1^ Pa^−1^ = 10^−10^ cm^3^(STP) cm cm^−2^ s^−1^ cmHg^−1^) [45]. The diffusion coefficients *D* were determined using the time-lag method. The solubility coefficients *S* were determined using the ratio *S* = *P/D*. Ideal selectivity of gas separation α for gases *i* and *j* were calculated as *α_ij_* = *P_i_/P_j_*. Diffusion selectivity *α^D^* for gases *i* and *j* were calculated as *α^D^_ij_ = D_i_/D_j_.* The measurement error for *P* was 5%, for *D*, it was 10%; for *S*, it was 15%; for *α*, it was 10%; and for *α^D^*, it was 20%. 

The solubility coefficient (*S_i_*), of a gas *i* in a membrane is the ratio between its solubility *C_i_* in the membrane and its fugacity *f_i_* or partial pressure *pi* in the gas phase at constant temperature [45]: *S_i_* = *C_i_f_i_*.

#### 2.4.9. AFM Studies

Surface topography of samples was imaged on an atomic force microscope (AFM) Nano-DST (Pacific Nanotechnology, Santa Clara, CA, USA), operated in semi-contact (tapping) mode under ambient conditions. Probes NSG01 (by TipsNano, Estonia) were used with the following parameters: n-Si, tip curvature 10 nm, frequency ca. 150 kHz and force constant 5.1 N/m.

## 3. Results and Discussion

### 3.1. The ASiP–AGM Characterization

**Figure 1:**
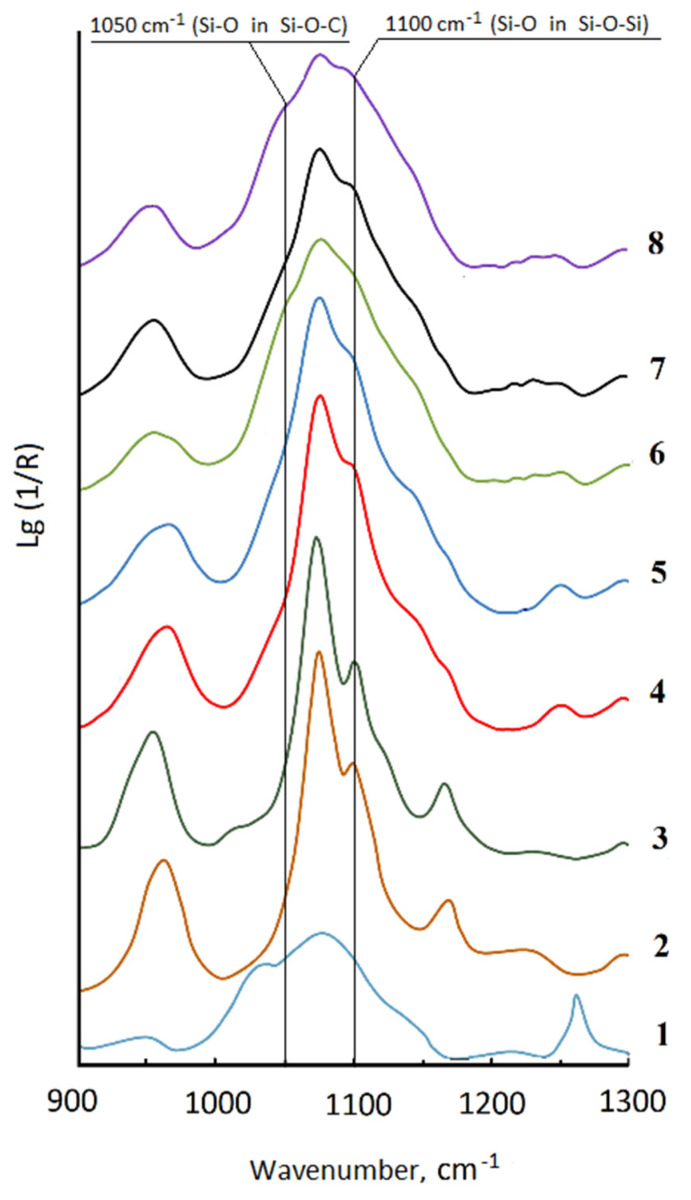
FTIR spectra of PEO (1), TEOS (2), AGM (3), PEOASiP (4), ASiP–AGM-10 (5), ASiP–AGM-50 (6), ASiP–AGM-80 (7) and ASiP–AGM-100 (8).

FTIR spectroscopy was used to investigate the structure of the ASiP–AGM-(10 ÷ 100). According to Figure 1, the ASiP–AGM-10 spectrum practically does not differ from the PEOASiP spectrum.

**Figure 2:**
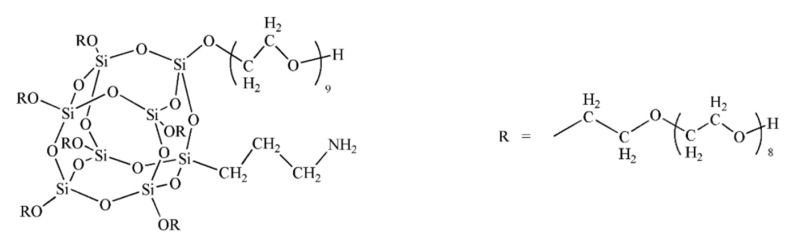
Silica of a cubic structure with an aminopropyl and PEO branches.

**Figure 3:**
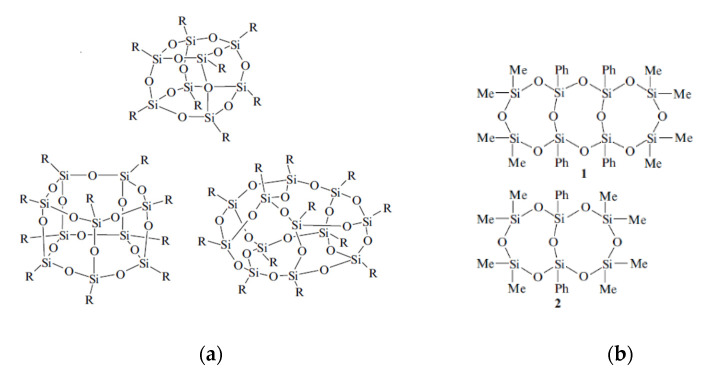
Structures of cyclic (**a**) and framework (**b**) silsesquioxane.

In previously work [43], the cubic structure of the ASiP was established by the NMR spectra. It can be concluded that the ASiP–AGM, obtained at a relatively low content of AGM, is a silica of a cubic structure with an aminopropyl and PEO branches (Figure 2):

With an increase of AGM content in the ASiP–AGM composition up to the complete replacement of TEOS by AGM, a shoulder appears and increases in the spectra on the region of 1050 cm^−1^. According to References [46,47], the presence of one broad band in the 1050–1100 cm^−1^ region is characteristic of cyclic (a) and framework (b) silsesquioxane structures (Figure 3).

**Figure 4:**
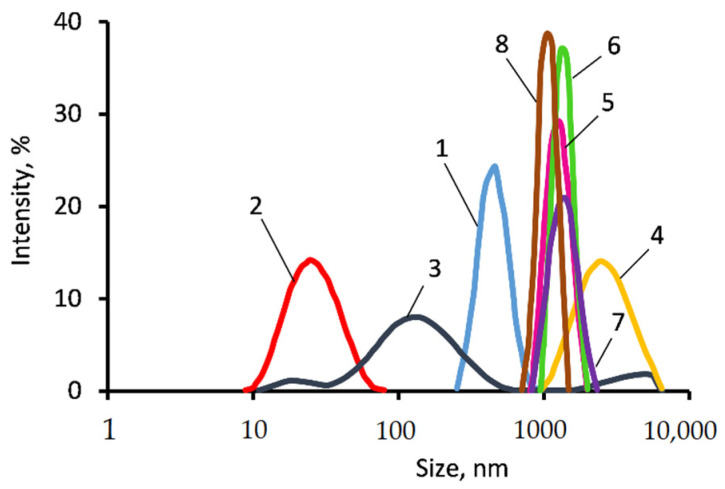
Particle size distribution in the environment of toluene for PEO (1), TEOS (2), PEOASiP (3), AGM (4), ASiP–AGM-10 (5), ASiP–AGM-30 (6), ASiP–AGM-50 (7) and ASiP–AGM-100 (8).

The ASiP–AGM particle size distribution was investigated using the dynamic light scattering method (Figure 4). It was found that AGM molecules form associates, and the sizes are in the range of 1000–6000 nm, with a maximum at 2500 nm. The tendency of the secondary amine to associative interaction leads to the formation of particles, the sizes are significantly larger than the sizes of the PEOASiP particles (Figure 4, line 3). A feature of the ASiP–AGM is the observed narrow particle size distribution compared to PEOASiP. In addition, regardless of the AGM content in the ASiP–AGM, the particle sizes are quite close in their values. Thus, the maximum particle size distribution for the ASiP–AGM-10 is at 1015 nm, the ASiP–AGM-30 is at 1044 nm, the ASiP–AGM-50 is at 1166 nm and the ASiP–AGM-100 is at 1152 nm.

**Figure 5:**
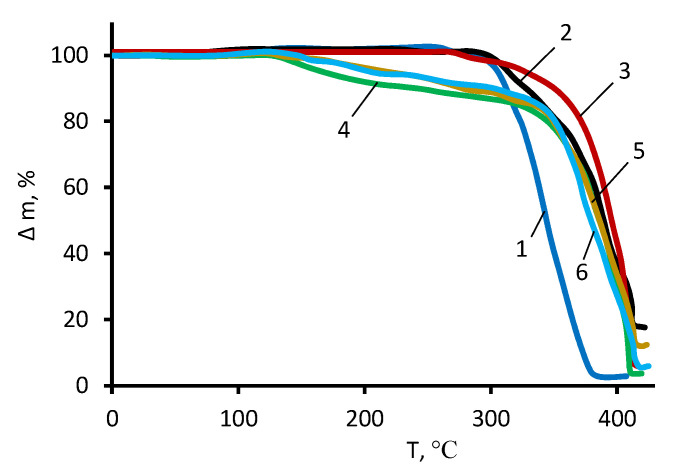
TGA of PEO (1), PEOASiP (2), ASiP–AGM-10 (3), ASiP–AGM-20 (4), ASiP–AGM-30 (5) and ASiP–AGM-50 (6).

The assumption that structure of the ASiP–AGM-10 is similar to the cubic structure of the PEOASiP is confirmed by the thermogravimetric analysis data (Figure 5). The TGA curves practically coincide and indicate a high thermal stability of the PEOASiP and the ASiP–AGM-10.

An increase of AGM content in the ASiP–AGM leads to a decrease in the thermal stability of these compounds. Temperature range at 170–300 °C; corresponds to the beginning of weight loss for the ASiP–AGM-20, the ASiP–AGM-30 and the ASiP–AGM-50. According to Reference [44], the onset of weight loss is in the range of 200–300 °C for cyclic silsesquioxanes. Weight loss for the ASiP–AGM-(20 ÷ 50) reaches 20% at 300 °C. Total thermal destruction of the ASiP–AGM-(20 ÷ 50) begins at a temperature of 350 °C. Based on the results, it can be assumed that the ASiP–AGM-(20 ÷ 50) is a mixture of cyclic and framework organosilicon structures.

**Figure 6:**
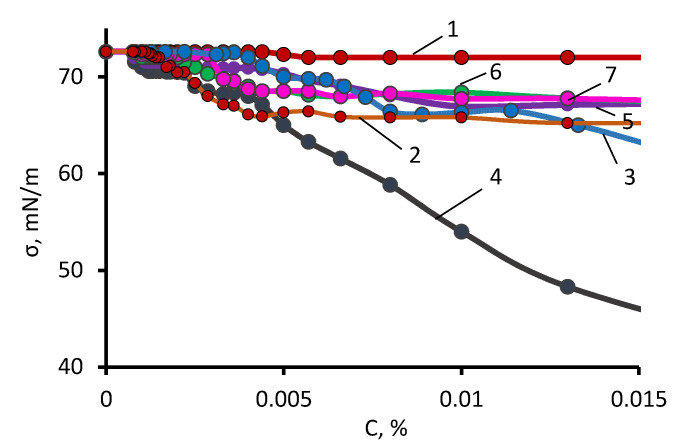
Isotherm curves of surface tension (σ, mN/m) on concentration in water (C, %) for PEO (1), AGM (2), PEOASiP (3), ASiP–AGM-10 (4), ASiP–AGM-50 (5), ASiP–AGM-80 (6) and ASiP–AGM-100 (7).

Surface tension isotherms of the ASiP–AGM changes depending on the content of AGM in their composition (Figure 6). The ASiP–AGM-10 has the lowest critical micelle concentration (CMC) and surface tension. The CMC values and surface tension of these compounds increase with an increase of AGM content in the ASiP–AGM. It should be noted that CMC for the ASiP–AGM-10 is lower compared to the CMC for the PEOASiP. Thus, the presence of a secondary amine, transformed in an aqueous medium into an ammonium cation, leads to an increase of the ASiP–AGM-10 amphiphilicity in comparison with the PEOASiP. An increase of the CMC, surface tension and a change of isotherm curves with an increase of AGM content in the ASiP–AGM is a consequence of differences in the ASiP–AGM-10 and the ASiP–AGM-(20 ÷ 100) structures. 

### 3.2. Investigation of MBC Modified by the ASiP–AGM

**Figure 7:**
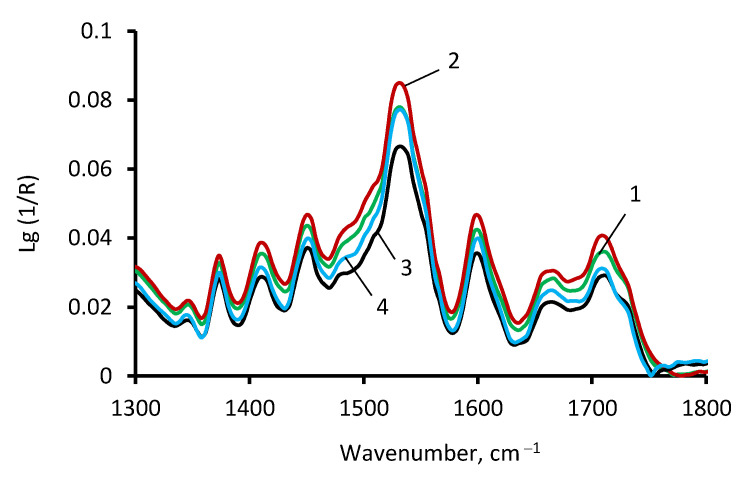
FTIR spectra of polymers modified with ASiP–AGM-10 at its content of 0 (1), 0.05 (2), 0.1 (3) and 0.2 (4) wt%.

The ASiP–AGM-10 was used as a modifier due to the most pronounced amphiphilicity. The possibility of O-polyisocyanates formation during the interaction of PPEG and TDI in the ASiP–AGM-10 presence was investigated by using FTIR spectroscopy (Figure 7). It was found that bands in the region of 1710 and 1410 cm^−1^ are practically absent in the FTIR spectra. It is typical for stretching vibrations of the C=O bond in the composition of polyisocyanurate structures. The analytical band is present at 1670 cm^−1^, due to stretching vibrations of the N=C bond in the composition of O-polyisocyanate structures. That means the modification of the reaction system based on PPEG and TDI, using the ASiP–AGM-10, does not prevent on the formation of O-polyisocyanate structures.

**Figure 8:**
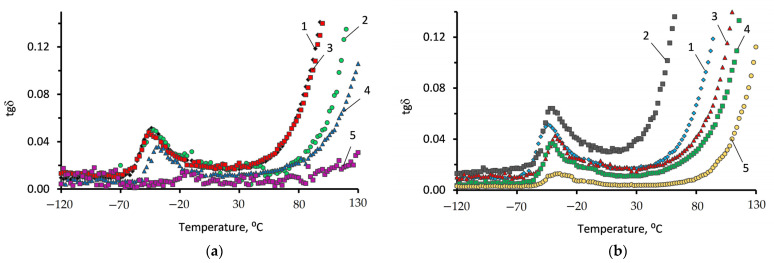
Temperature DLT dependences of polymers modified by the ASiP–AGM-10 (**a**) and the ASiP–AGM-50 (**b**) with their content: 0.0 (1), 0.05 (2), 0.10 (3), 0.20 (4) and 0.30 (5) wt.%.

To study the supramolecular structure of the modified polymers, the temperature dependences of the dielectric loss tangent (DLT) were measured. It was found that the ASiP–AGM-10 (Figure 8a) has a significant effect on α-transitions temperature of polymers in the region of its low content. For an unmodified polymer, the region of the α-transition is observed at T = −47 °C. At 0.05 wt.% of the ASiP–AGM-10, there aren’t noticeable changes in the temperature of α-transition, but relaxation transitions in the high-temperature region are shifted from 50 to 80 °C. It can be concluded that polymer modified with 0.05 wt.% of the ASiP–AGM-10 becomes denser.

The sample modified with 0.10 wt.% of the ASiP–AGM-10 has high-temperature relaxation transitions similar to the control sample. However, in this case, the second α-transition occurs at −35 °C with the α-transition at −47 °C. The detected difference in the temperature range of α-transitions is a consequence of the polyoxypropylene and polyoxyethylene segments microphase separation.

At 0.20 wt.% of the ASiP–AGM-10 content the α-transition region shifts to −35 °C and the curve becomes flatter in the high-temperature relaxation transition region. A further increase of the ASiP–AGM-10 content to 0.30 wt.% leads to a shift in the α-transition region to −10 °C. The results indicate the active effect of the ASiP–AGM-10 on the supramolecular structure of block copolymers.

The ASiP–AGM-50 was used instead of the ASiP–AGM-10 to evaluate the role of the ASiP–AGM chemical structure in its modifying ability (Figure 8b). The ASiP–AGM-50 is a mixture of organosilicons of cyclic structure in contrast to the ASiP–AGM-10 of cubic structure. According to the temperature dependences of the DLT, when the ASiP–AGM-50 is used as a modifier, the main changes are observed at high temperatures and are not additive. An increase in the temperature of relaxation transitions with a change of the ASiP–AGM-50 content indicates that polymer becomes denser.

**Figure 9:**
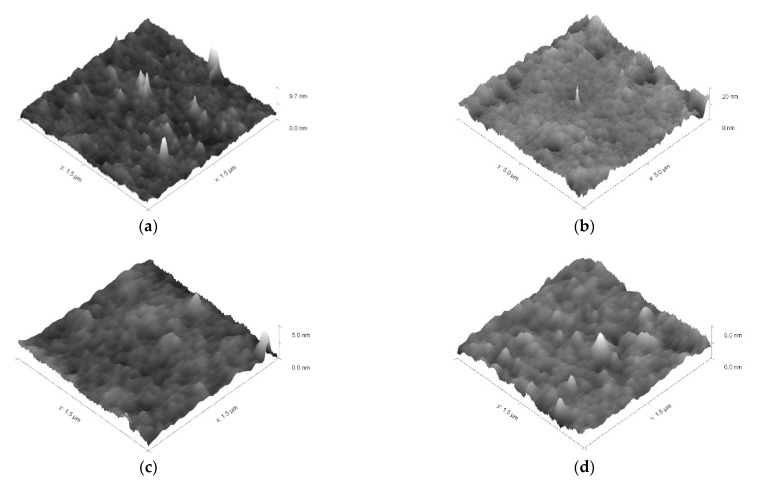
AFM images of polymers modified by [ASiP–AGM-10] = 0 (**a**), 0.1 (**b**), 0.2 (**c**) and 0.3 (**d**) wt.%.

The film morphology was investigated by using AFM (Figure 9). The AFM images of the surface topography show multiple repeating relief features that are rounded. Relatively equal sizes and uniform distribution of relief elements indicate the formation of segregated structures here. The use of a modifier in the synthesis of polymers leads to a change in the AFM image.

### 3.3. Mechanical Behavior of Modified Polymers

**Figure 10:**
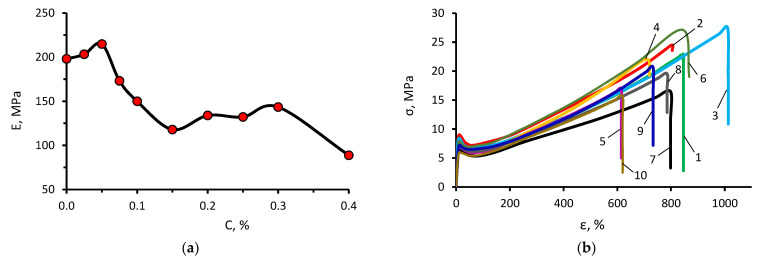
Dependence of Young’s modulus (E, MPa) of polymers on the content of the ASiP–AGM-10 in their composition (**a**) and tensile tests (**b**) of polymers obtained at [ASiP–AGM-10] = 0.0 (1), 0.025 (2), 0.05 (3), 0.075 (4), 0.10 (5), 0.15 (6), 0.20 (7), 0.25 (8), 0.30 (9), 0.40 and (10) wt.%.

Young’s modulus (Figure 10a) and stress–strain curves (Figure 10b) of polymers reflect the non-additive effect of the ASiP–AGM-10 content on their mechanical behavior.

### 3.4. Investigation of the Sorption Activity of Modified Polymers

**Figure 11:**
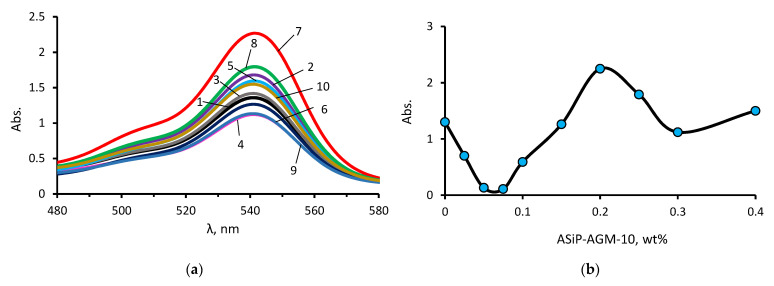
Spectra of Rhodamine 6G, sorbed on polymers obtained at [ASiP–AGM-10] = 0.0 (1), 0.025 (2), 0.05 (3), 0.075 (4), 0.10 (5), 0.15 (6), 0.20 (7), 0.25 (8), 0.30 (9) and 0.40 (10) wt.% (**a**); and the dependence of absorption coefficient (Abs, λ = 535 nm) on the content of the ASiP–AGM-10 (**b**).

The dependence of the sorption activity of polymers on the ASiP–AGM-10 content was studied using the dye Rhodamine 6G. The spectrum of Rhodamine 6G sorbed on both the initial and modified polymer shifts from 535 to 545 nm of the absorption maximum (Figure 11). Thus, the inner cavity of the voids that determine the free volume of the polymers is active in relation to the dye molecules. The result of the modifying effect most likely depends on the ratio of polyoxyethylene and polyoxypropylene segments on the inner surface of the voids and their free volume changes. The non-additive dependence of the sorption coefficient on the ASiP–AGM-10 content is a consequence of its active influence on the supramolecular structure of the polymer. Similar to the dependence of Young’s modulus on the ASiP–AGM-10 content (Figure 10a), the sorption capacity of polymers is non-additive. Moreover, growth areas of the Young’s modulus values coincide with the area of the decrease in the sorption capacity in contrast.

### 3.5. Gas Transport Properties of MBC Modified with the ASiP–AGM-10 and the ASiP

The obtained polymers were investigated as gas permeable membranes. According to the results (Table 1), the gas permeability (*P*) of polymers weakly depends on the modifier content.

Nevertheless, at 0.10 wt.% of the ASiP–AGM-10 content, the permeability coefficient for oxygen and nitrogen decreases compared with the initial polymer. At 0.20 wt.% of the ASiP–AGM-10 content, an increase of *P* is observed, it is outside the error limits for all gases except oxygen.

The gas permeability of the polymers modified with the ASiP is slightly lower than the permeability of the original polymer. The ideal gas-separation selectivity (*α* = *P_i_/P_j_* for different gas pairs *i* and *j*) is shown in Table 2.

According to References [13,45,48], the O_2_/N_2_ pair is characterized by low values of the selectivity of gas separation (α = 2.5 ÷ 3) for rubbers. However, polymers have maximum selectivity (α = 39) for gas pair containing nitrogen at 0.10 wt.% of the ASiP–AGM-10. This is due to a decrease of nitrogen gas permeability in this material (Table 1). Changes of the ideal selectivity for gas pairs containing methane are not observed. These properties are less pronounced for the ASiP modifier compared to the ASiP–AGM-10. Thus, at 0.10 wt.% of the ASiP content, the selectivity of gas pairs containing nitrogen is higher than for the initial polymer. Selectivity becomes close to the initial polymer with increasing of the ASiP content.

An increase of the ideal selectivity of the CO_2_/N_2_ gas pair (from 25 to 39) by 1.5 times, observed at 0.10 wt.% of the ASiP–AGM-10, demonstrates the promising use of this polymer material for flue gases separation.

The diffusion coefficients (*D*) of oxygen, nitrogen, carbon dioxide and methane are given in Table 3. The diffusion coefficients for helium and hydrogen were not calculated due to extremely short delay times.

According to the results (Table 3), an increase of the permeability coefficient is accompanied by a slight increase of *D* at 0.20 wt.% of the ASiP–AGM-10 content. A decrease of *D* compared with the initial polymer is observed at 0.10 wt.% of the ASiP–AGM-10 content. The use 0.10 wt.% of the ASiP modifier leads to a slight increase of gas diffusion coefficient. Consequently, permeability changes are associated with changes of diffusion coefficients. However, these changes are small and in fact are within the measurement error. The diffusion selectivity *α^D^* = *D_i_/D_j_* (for different gas pairs *i* and *j*) also does not change: for gases pair O_2_/N_2_, *α^D^* is 1.4 ± 0.2; for gases pair CO_2_/N_2_, it is 0.7 ± 0.1; and for gases pair CO_2_/CH_4_, it is 1.1 ± 0.1. In contrast to glassy polymers [49], the features of the supramolecular chain packing in this case do not affect the gas transport properties of the studied polymers. 

The gas solubility coefficients (*S*) practically do not changes with the modifier content (Table 4). In contrast to glassy polymers [45,49], the solubility of gases in rubber polymers does not related with the peculiarity of the supramolecular structure. It can be concluded that changes of gas permeability are associated only with changes of diffusion coefficients. From the results obtained, it can be concluded that the effect of ASiP–AGM-10 on the properties of polymers, including solubility and diffusion coefficients, is associated with a modifying effect on the supramolecular structure of polymers.

## 4. Conclusions

The ASiP–AGM was synthesized by consistent exchange of tetraethoxysilane with AGM in the PEOASiP. The assumption that the ASiP–AGM-10 structure is similar to the PEOASiP cubic structure was confirmed by FTIR spectroscopy and thermogravimetric analysis. An increase in the AGM content from 20 to 100 wt.% leads to the predominant formation of a mixture of cyclic and framework organosilicon structures. The presence of a secondary amine, converted in an aqueous medium into an ammonium cation, leads to an increase of the ASiP–AGM-10 amphiphilicity compared with the PEOASiP.

ASiP–AGM has been used as a modifier of block copolymers, and the peculiarity is the presence in its structure of coplanar rigid polyisocyanate blocks of acetal nature (O-polyisocyanates). It was found that modification of the reaction system based on PPEG and TDI, using the ASiP–AGM-10, does not prevent the formation of O-polyisocyanate structures.

According to the temperature dependences of the dielectric loss tangent, the ASiP–AGM-10 has a non-additive effect on high-temperature relaxation and α-transitions of modified polymers. Young’s modulus and stress–strain curves of polymers reflect the non-additive effect of the ASiP–AGM-10 content on their mechanical behavior. Growth areas of the Young’s modulus values coincide with the area of the decrease in the sorption capacity in contrast. The results indicate the ability of the ASiP–AGM-10 to influence the supramolecular structure of block copolymers.

Modified block copolymers were investigated as gas transport membranes. The increase of the permeability coefficients is due to the increase of the diffusion coefficients. However, the gas solubility coefficients do not change. The growth in the ideal selectivity for a number of gas pairs is observed. An increase of the ideal selectivity for the CO_2_/N_2_ gas pair (from 25 to 39) by 1.5 times, observed at 0.10 wt.% of the ASiP–AGM-10, demonstrates the promising use of this polymer material for flue gases separation.

## Figures and Tables

**Table 1:** Permeability coefficients (*P*).

Modifier		ASiP–AGM-10				ASiP		
C, %	0.0	0.05	0.10	0.20	0.30	0.10	0.20	0.30
Gas	*P*, Barrer *							
He	13 ± 0.7	12 ± 0.6	12 ± 0.6	14 ± 0.7	12 ± 0.6	11 ± 0.6	11 ± 0.6	12 ± 0.6
H_2_	17 ± 0.9	19 ± 1	19 ± 1	22 ± 1	18 ± 0.9	15 ± 0.8	16 ± 0.8	17 ± 0.9
O_2_	9.0 ± 0.5	9.8 ± 0.5	8.4 ± 0.4	10 ± 0.5	8.3 ± 0.4	8.5 ± 0.4	7.8 ± 0.4	7.8 ± 0.4
N_2_	3.2 ± 0.2	3.4 ± 0.2	2.2 ± 0.1	4.0 ± 0.2	3.2 ± 0.2	2.7 ± 0.1	2.7 ± 0.1	2.9 ± 0.1
CO_2_	82 ± 4	85 ± 4	85 ± 4	110 ± 6	84 ± 4	86 ± 4	78 ± 4	86 ± 4
CH_4_	9.9 ± 0.5	10 ± 0.5	10 ± 0.5	13 ± 0.7	10 ± 0.5	11 ± 0.6	9.0 ± 0.5	0.6

* 1 Barrer =10^−10^·cm^3^ (STP) cm/(cm^2^·s·cmHg).

**Table 2:** Ideal selectivity for gas pairs (*α* *= P_i_/P_j_*).

Modifier		ASiP–AGM-10				ASiP		
C, %	0.0	0.05	0.10	0.20	0.30	0.10	0.20	0.30
Gas pairs	*α* = *P_i_/P_j_*							
O_2_/N_2_	2.8 ± 0.3	2.9 ± 0.3	3.8 ± 0.4	2.5 ± 0.3	2.6 ± 0.3	3.1 ± 0.3	2.9 ± 0.3	2.7 ± 0.3
CO_2_/N_2_	26 ± 3	25 ± 3	39 ± 4	28 ± 3	26 ± 3	32 ± 3	29 ± 3	30 ± 3
He/N_2_	4.1 ± 0.4	3.5 ± 0.4	5.5 ± 0.6	3.5 ± 0.4	3.8 ± 0.4	4.1 ± 0.4	4.1 ± 0.4	4.1 ± 0.4
H_2_/N_2_	5.3 ± 0.5	5.4 ± 0.5	8.6 ± 0.9	5.5 ± 0.6	5.6 ± 0.6	8.6 ± 0.9	5.5 ± 0.6	5.6 ± 0.6
CO_2_/CH_4_	8.2 ± 0.8	8.5 ± 0.9	8.5 ± 0.9	8.5 ± 0.9	8.4 ± 0.8	7.8 ± 0.8	8.7 ± 0.9	7.8 ± 0.8
He/CH_4_	1.3 ± 0.1	1.2 ± 0.1	1.2 ± 0.1	1.1 ± 0.1	1.2 ± 0.1	1.0 ± 0.1	1.2 ± 0.1	1.1 ± 0.1
H_2_/CH_4_	1.7 ± 0.2	1.9 ± 0.2	1.9 ± 0.2	1.7 ± 0.2	1.8 ± 0.2	1.4 ± 0.1	1.8 ± 0.2	1.5 ± 0.2

**Table 3:** Diffusion coefficients (D).

Modifier		ASiP–AGM-10				ASiP		
C, %	0.0	0.05	0.10	0.20	0.30	0.10	0.20	0.30
Gas	*D*·10^8^, cm^2^/s							
O_2_	94 ± 9	96 ± 10	85 ± 9	110 ± 10	85 ± 9	110 ± 10	90 ± 9	95 ± 10
N_2_	67 ± 7	79 ± 8	66 ± 7	100 ± 10	74 ± 7	64 ± 6	57 ± 6	79 ± 8
CO_2_	44 ± 4	45 ± 5	45 ± 5	66 ± 7	47 ± 5	48 ± 5	48 ± 5	47 ± 5
CH_4_	39 ± 4	41 ± 4	41 ± 4	58 ± 4	42 ± 4	45 ± 5	45 ± 5	47 ± 5

**Table 4:** Solubility coefficients (*S*).

Modifier		ASiP–AGM-10				ASiP		
C, %	0.0	0.05	0.10	0.20	0.30	0.10	0.20	0.30
Gas	*S*·10^3^, cm^3^(STP)/(cm^3^·cmHg)							
O_2_	1.0 ± 0.1	1.0 ± 0.2	1.0 ± 0.2	0.9 ± 0.1	1.0 ± 0.2	0.8 ± 0.1	0.9 ± 0.1	0.8 ± 0.1
N_2_	0.5 ± 0.1	0.4 ± 0.1	0.3 ± 0.1	0.4 ± 0.1	0.4 ± 0.1	0.4 ± 0.1	0.5 ± 0.1	0.4 ± 0.1
CO_2_	19 ± 3	19 ± 3	19 ± 3	17 ± 3	18 ± 3	18 ± 3	16 ± 2	18 ± 3
CH_4_	2.5 ± 0.4	2.4 ± 0.4	2.4 ± 0.4	2.2 ± 0.3	2.4 ± 0.4	2.4 ± 0.4	2.0 ± 0.3	2.3 ± 0.3

## Abbreviations

PPEG	block copolymer of propylene and ethylene oxide
TDI	2,4-tolylene diisocyanate
ASiP	silica branched by polyoxyethylene oxide and PDMS
ASiP–AGM-(10 ÷ 100)	silica branched by polyoxyethylene oxide and AGM-(10 ÷ 100 wt.% of TEOS total amount)
PEOASiP	silica branched by polyoxyethylene oxide
PEO	polyoxyethylene oxide
TEOS	tetraethoxysilane
DEG-K	potassium diethylene glycolate
AGM	3-aminopropyltriethoxysilane
PDMS	polydimethylsiloxane (MW ≈ 30,000 g/mol)
TMA	thermomechanical analysis
TGA	thermogravimetric analysis
DLT	dielectric loss tangent
FTIR	Fourier transform infrared spectroscopy analysis
CMC	critical micelle concentration
